# Fibronectin is a stress responsive gene regulated by HSF1 in response to geldanamycin

**DOI:** 10.1038/s41598-017-18061-y

**Published:** 2017-12-15

**Authors:** Karim Colin Hassan Dhanani, William John Samson, Adrienne Lesley Edkins

**Affiliations:** Biomedical Biotechnology Research Unit (BioBRU), Department of Biochemistry and Microbiology, Rhodes University, Grahamstown, 6140 South Africa

## Abstract

Fibronectin is an extracellular matrix glycoprotein with key roles in cell adhesion and migration. Hsp90 binds directly to fibronectin and Hsp90 depletion regulates fibronectin matrix stability. Where inhibition of Hsp90 with a C-terminal inhibitor, novobiocin, reduced the fibronectin matrix, treatment with an N-terminal inhibitor, geldanamycin, increased fibronectin levels. Geldanamycin treatment induced a stress response and a strong dose and time dependent increase in fibronectin mRNA via activation of the fibronectin promoter. Three putative heat shock elements (HSEs) were identified in the fibronectin promoter. Loss of two of these HSEs reduced both basal and geldanamycin-induced promoter activity, as did inhibition of the stress-responsive transcription factor HSF1. Binding of HSF1 to one of the putative HSE was confirmed by ChIP under basal conditions, and occupancy shown to increase with geldanamycin treatment. These data support the hypothesis that fibronectin is stress-responsive and a functional HSF1 target gene. COLA42 and LAMB3 mRNA levels were also increased with geldanamycin indicating that regulation of extracellular matrix (ECM) genes by HSF1 may be a wider phenomenon. Taken together, these data have implications for our understanding of ECM dynamics in stress-related diseases in which HSF1 is activated, and where the clinical application of N-terminal Hsp90 inhibitors is intended.

## Introduction

Tissue integrity is maintained by the attachment of cells to a network of secreted proteins, proteoglycans, glycoproteins and polysaccharides known as the extracellular matrix (ECM). Fibronectin (FN) was the first matrix glycoprotein to be extensively studied, and is a ubiquitous ECM component produced by almost all cell types^1^. In humans, FN is encoded by a single gene (*FN1*) on chromosome 2. The protein coding region of the *FN1* gene consists of 46 exons and the primary transcript is alternately spliced to create at least 20 splice variants^2^. FN is a multi-domain glycoprotein found intracellularly as a soluble 450 kDa disulphide-linked dimer, and in the extracellular matrix as large insoluble multimers. FN binds cell surfaces and a number of other ECM components including collagen, fibrin, heparin and integrins^3^. FN is secreted by the cell as a soluble dimer which is later rendered insoluble through conformational changes initiated through integrin interactions^4^.

FN is involved in cell adhesion and migration processes including embryogenesis, wound healing, blood coagulation, host defence, the maintenance of cell shape and opsonisation^5–8^. FN-receptor interactions play an important role in tumour cell biology^1,9^ and in the progression of fibrosis^10^, synovial related diseases^11^ and even Alzheimer’s disease^12^. In cancer, high FN levels are associated with increased invasion and metastatic capability in lung cancers and hepatic cancers^9,13^. In other cancer types, however, low levels of FN expression have been found to correlate with increased migratory capacity^14^. Some authors have proposed that the deposition of ECM proteins such as collagen and FN act as a barrier to the growth of tumours^15^, while circulating FN has been shown to be required for tumour growth and angiogenesis^16^. In fibrosis and inflammation related diseases, the increased deposition of ECM components including FN is known to be a causative factor in the development of pathological conditions such as cirrhosis of the liver and Crohn’s disease^17–19^.

Given that changes in the expression level of FN are linked to pathology, an understanding of the conditions that regulate FN levels is important. The *FN1* promoter has been described and the −170 bp CRE and the −150 CAAT elements shown to act as a single functional element within the core promoter leading to cell line specific FN expression patterns^20^. Expression of FN is induced by stimuli including dexamethasone^21^, IL-4^22^, TGF-β^23^ and forskolin^24^. Dexamethasone leads to increased FN mRNA stability, while TGF-β treatment led to activation of β-catenin and stimulation of the FN promoter via functional LEF-TCF sites identified in the FN promoter^25^. Forskolin causes a similar increase in *FN1* promoter activity but via activation of adenylate cyclase acting through the CRE element at positions −188 bp to −157 of the gene. *FN1* expression is also known to be regulated by COX-2 and sphingosine-1-phosphate (SP1) although the mechanisms are not clearly defined^26,27^. In contrast, oncogenic transformation with RAS has been shown to reduce the expression of FN^28^.

Previous work by our group identified a direct interaction between FN and Hsp90^29^. Hsp90 is a molecular chaperone that stabilises intermediate conformations of a large number of important cellular proteins, known as client proteins^30,31^. Decreased levels of both total and extracellular FN matrix were observed upon treatment of cells with the C-terminal Hsp90 inhibitor, novobiocin (NOV), and upon depletion of Hsp90 by RNA interference. The effects of NOV or Hsp90 knock-down could be rescued by extracellular Hsp90^29^. In the course of this work it was noted that another inhibitor of Hsp90, geldanamycin (GA), did not produce the same FN matrix phenotype observed with either NOV treatment or knock-down of Hsp90. Hsp90 knock-down and NOV treatment reduced the FN matrix, while GA treatment appeared to cause an increase in the levels of the FN ECM. GA is an N-terminal inhibitor of Hsp90 and the first naturally occurring Hsp90 inhibitor to be studied^32^. Analogues of GA form the first generation of anti-Hsp90 inhibitors to be assessed in clinical trials^33,34^. GA binds to the N-terminal ATP binding site of Hsp90, leading to inhibition of the chaperone and degradation of Hsp90 client proteins^35,36^. In addition, GA, along with a other N-terminal inhibitors of Hsp90, also results in the activation of the stress response^37^. This stress response is a coordinated series of molecular events that culminates in the changes in expression of stress-responsive genes through the activation of the transcription factor heat shock factor 1 (HSF1)^38^. Based on our observations of increased FN in response to GA treatment, and the fact that GA is known to induce a stress response, we investigated whether *FN1* was a stress responsive gene.

## Results

### Geldanamycin, but not novobiocin, increases FN levels

**Figure 1:**
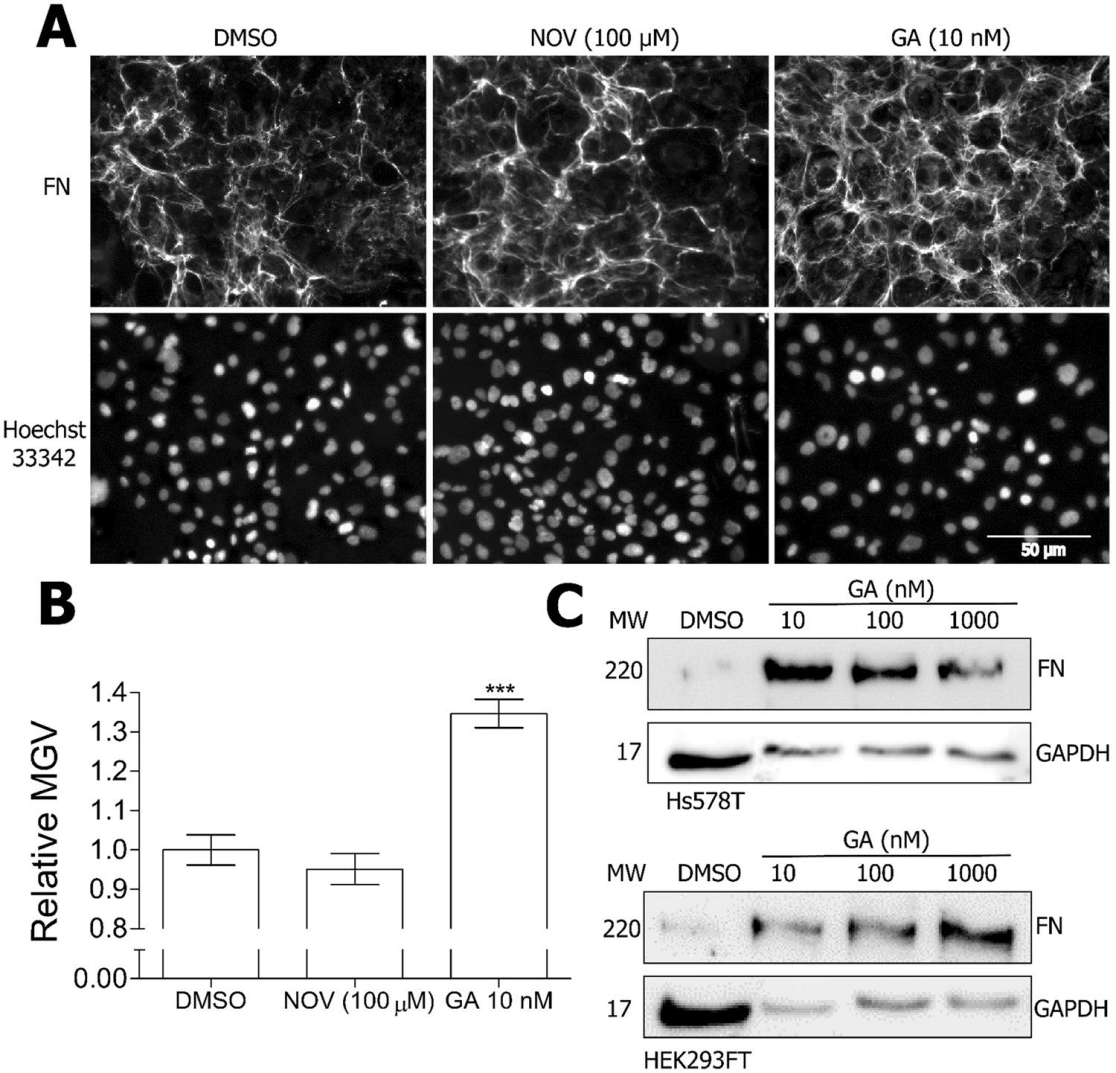
Geldanamycin treatment increases fibronectin extracellular matrix and protein levels. (**A**) Hs578T cells were treated with 100 µM NOV or 10 nM GA or DMSO before immunostaining with mouse anti-human FN followed by donkey anti-mouse DyLight 488 secondary antibody. Nuclei were stained with Hoechst 33342 (1 µg.mL^−1^). Images were captured using the 20 × objective on a Zeiss Axio Vert.A1 fluorescence microscope and analysed using ZEN Blue SP1 (Zeiss, Germany). The scale bar indicates 50 µm. (**B**) Average mean grey values (MGV) of FN staining in Hs578T cells from 6 independent fields of view from duplicate independent experiments were calculated in ImageJ. Statistical analysis was performed by comparing inhibitor treated to DMSO treated cells using one way ANOVA with Bonferroni post-test (***p* < *0.001). (**C**) Hs578T and HEK293FT cells were treated with different concentrations of GA or DMSO before levels of FN protein were analysed by immunoblotting. GAPDH was used as a loading control. Note that more lysate was loaded in the untreated samples than the GA-treated samples in order to detect the lower levels of basal FN expression without over saturation of the GA-treated signals.

**Figure 2:**
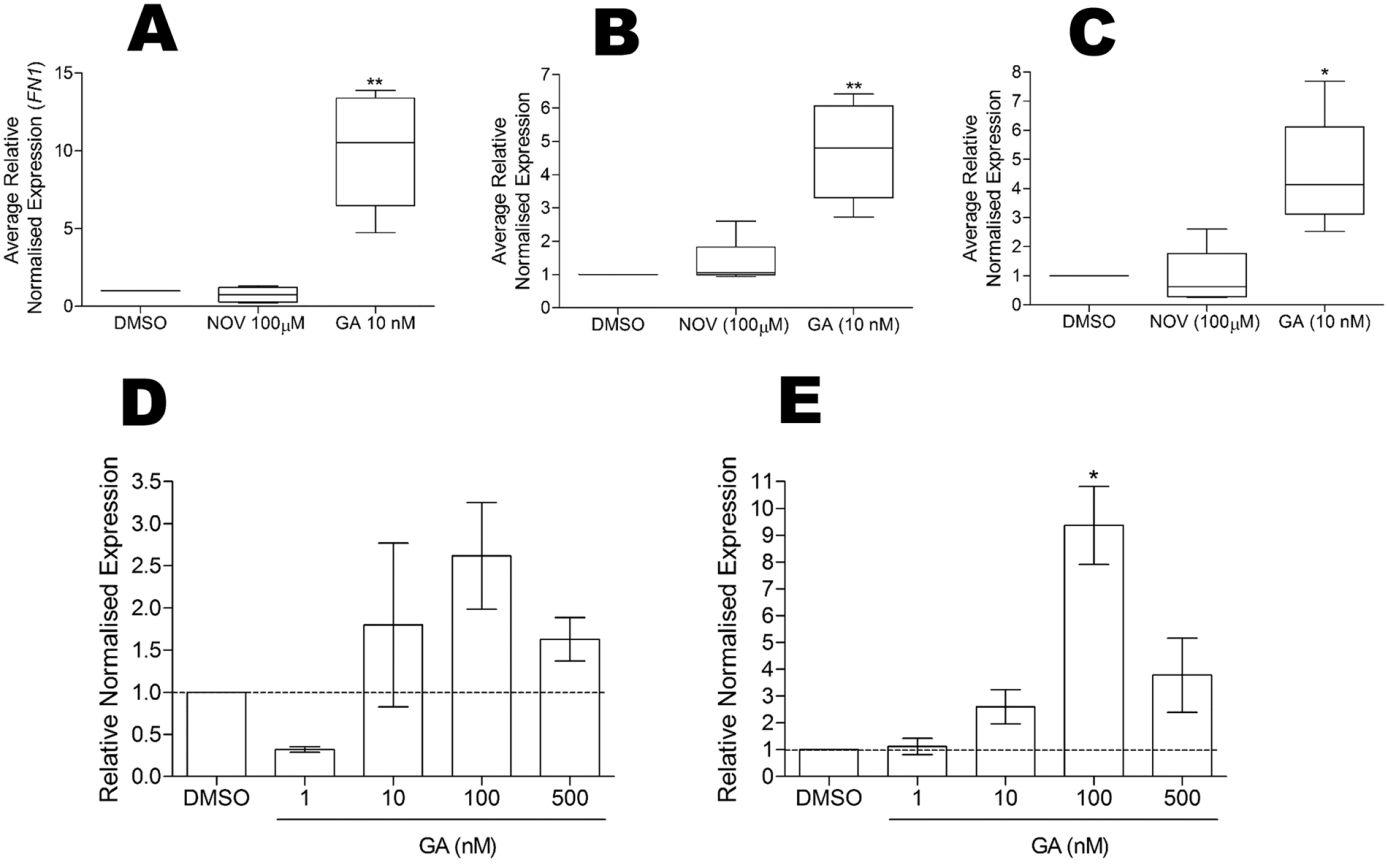
FN mRNA levels are increased by geldanamycin treatment. Hs578T cells were treated with 100 µM NOV, 10 nM GA or DMSO and the normalised expression (2^-ΔΔCt^) relative to the DMSO control calculated for (**A**) total FN, (**B**) EDA + containing FN and (**C**) EDB + containing FN. Averaged normalised expression values are from 4 independent biological replicates each with 3 technical replicates. (**D**) Hs578T and (E) HEK293FT cells were treated for 24 hours with different doses of DMSO or GA and normalised FN expression (2^-ΔΔCt^) relative to the DMSO control was calculated. Values are representative of duplicate experiments each conducted in triplicate. Statistical analysis comparing the inhibitor treated samples to the DMSO treated samples was conducted using a one way ANOVA with Bonferroni post-test (**p* < *0.01, *p < 0.05).

We previously demonstrated that FN interacts with and is stabilised by the molecular chaperone Hsp90^29^. During that study, we noticed a differential response in FN to inhibition of Hsp90 with an N-terminal versus C-terminal Hsp90 inhibitor. In order to investigate the effects of Hsp90 inhibition on the FN extracellular matrix (ECM), Hs587T cells (which constitutively express high levels of FN ECM in culture) were treated with subtoxic concentrations (data not shown) of the Hsp90 inhibitors novobiocin (NOV; C-terminal Hsp90 inhibitor) and geldanamycin (GA; N-terminal Hsp90 inhibitor) or the vehicle control, DMSO. GA treatment resulted in an increase in the levels of FN ECM in Hs578T cells (Fig. 1A,B), and a dose dependent increase in total FN protein in both Hs578T and HEK293T cells (Fig. 1C) compared to the vehicle control. In order to determine whether the observed increase in FN protein was related to changes in protein stability or mRNA abundance, the levels of FN mRNA were analysed (Fig. 2). GA treatment resulted in a statistically significant increase in total FN mRNA levels compared to the DMSO control (Fig. 2A). NOV treatment did not have any significant effect on total FN mRNA levels (Fig. 2A). Fibronectin is a highly spliced gene, containing three alternatively spliced exons which give rise to twenty distinct isoforms of the fibronectin protein. In addition to increases in total FN mRNA, we determined that GA caused an increase in the levels of EDA-containing (EDA +; Fig. 2B) and EDB-containing (EDB +; Fig. 2C) FN mRNA transcript splice variants. This, however, was not due to alterations in FN mRNA splicing, which remained unchanged in the presence and absence of GA (data not shown). Similar to total FN mRNA, NOV treatment had no significant effect on the levels of EDA + or EDB + containing FN mRNA. The increase in the levels of total FN mRNA in response to GA was determined to be dose-dependent in both Hs578T (Fig. 2D) and HEK293T (Fig. 2E) cell lines.

**Figure 3:**
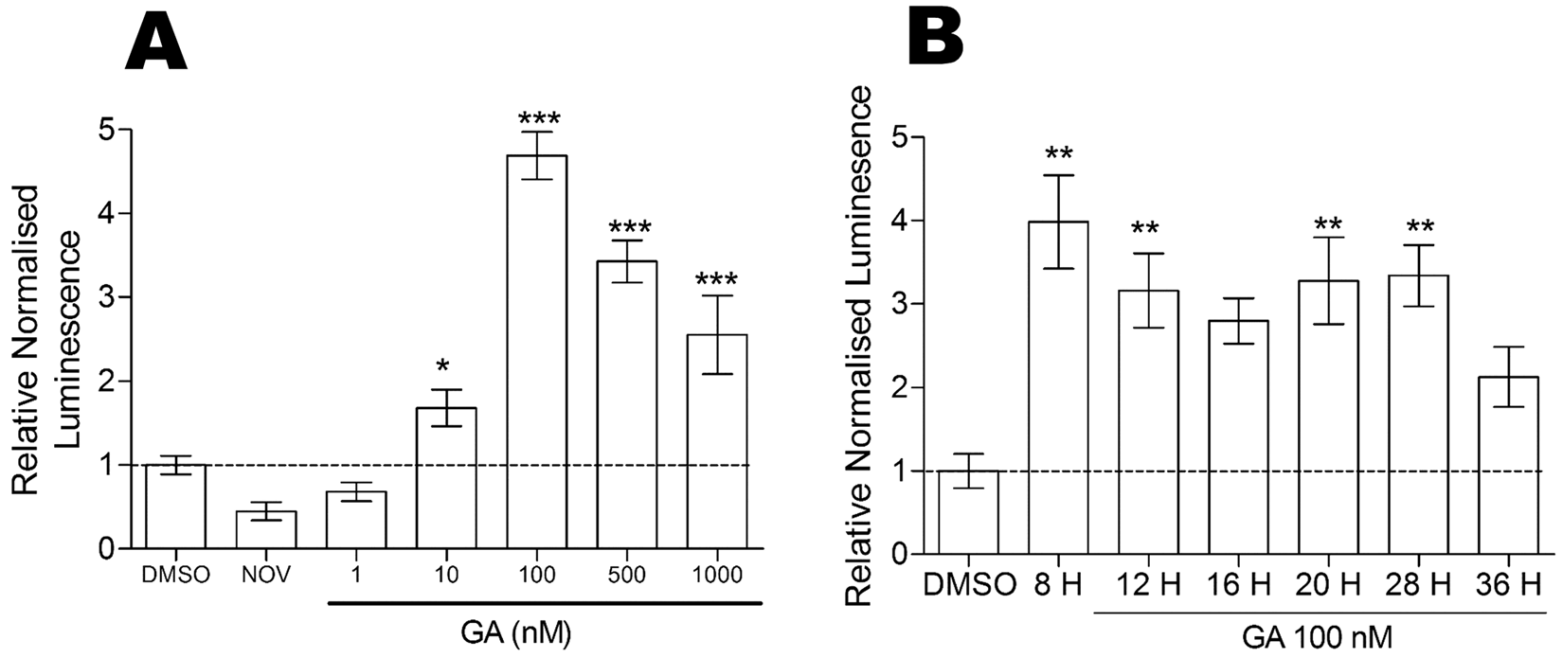
*FN1* promoter activity is increased by GA treatment. (**A**) HEK293FT cells transfected with pGL4-pFN1 promoter reporter and the transfection efficiency control pCAG-HRP-TM were treated with varying doses of GA, 100 µM NOV or DMSO. Relative luciferase activity values shown are representative of 12 biological replicates per treatment with 3 technical replicates per transfection. (**B**) HEK293FT cells transfected with pGL4-pFN1 reporter and pCAG-HRP-TM were treated with 100 nM GA for 8–36 hours. Relative luciferase activity values shown are representative of 3 independent transfections conducted in triplicate. Statistical analysis was conducted by comparing the inhibitor treated cells to the DMSO control using one way ANOVA with Bonferroni post-test (***p* < *0.001, **p < 0.01, *p < 0.05).

We next tested whether the increase in FN mRNA was due to increased promoter activity (Fig. 3). We cloned the region encompassing the *FN1* promoter (−2000 bp to + 1 of the *FN1* gene) upstream of a luciferase reporter construct to generate the *FN1* promoter luciferase reporter plasmid, pGL4-pFN1. GA treatment resulted in a statistically significant, dose-dependent increase in FN promoter activity in HEK293T cells compared to the DMSO treated cells (Fig. 3A). NOV treatment, consistent with the results from the mRNA analysis, had no effect on the *FN1* promoter (Fig. 3A). The kinetic analysis of promoter activation indicated that promoter activity increased within 8 hours of GA treatment and persisted for up to 36 hours post-treatment (Fig. 3B). Taken together, these results demonstrate that GA increases FN by increasing promoter activity leading to an increase in mRNA and subsequently FN protein.

### FN is a stress-responsive gene

**Figure 4:**
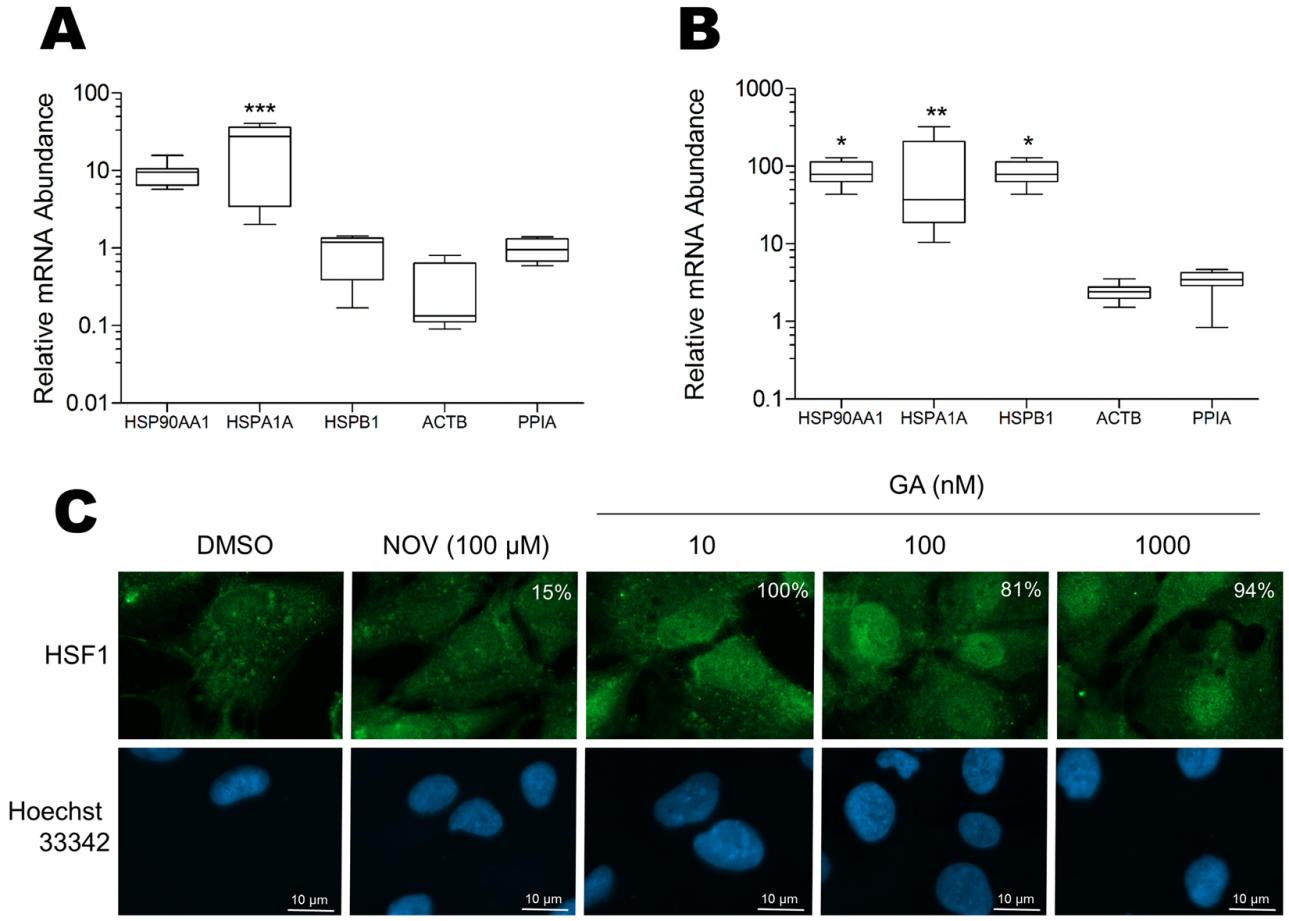
Geldanamycin treatment induces the heat-shock response. (**A**) Hs578T and (**B**) HEK293FT cells were treated with 100 nM GA or DMSO and the levels of stress-responsive genes (*HSP90AA1*, *HSPA1A*, *HSPB*) assessed by qRT-PCR using *PPIA* and *ACTB* reference genes. Data shown are average mRNA abundance (ΔCt) for genes in the GA treated sample relative to the DMSO treated control (which is 1 in all cases), calculated from 3 separate biological replicates each with 3 technical replicates. Statistical analysis was conducted compared to the *ACTB* reference gene using one way ANOVA with a Bonferroni post-test (***p* < *0.001, **p < 0.01, *p* < *0.05). (**C**) Hs578T cells were treated with DMSO, 100 µM novobiocin (NOV) or different concentrations of GA and immunostained for HSF1. Nuclei were stained with Hoechst33342 (1 µg.mL^−1^). The white numbering on the HSF1 panels indicates the percentage of cells with nuclear HSF1 in a minimum of three independent images. Images were captured using the 100× objective on a Zeiss Axio Vert.A1 fluorescence microscope and analysed using ZEN Blue SP1. Scale bar is 10 µm.

GA and other N-terminal Hsp90 inhibitors are known to induce a stress response, while C-terminal Hsp90 inhibitors like NOV do not. For the purposes of this study, we define the stress response to be that which leads to activation of heat shock factor (HSF) transcription factors that translocate to the nucleus and alter transcription of stress-responsive genes. The main transcription factor involved in regulation of the stress response is HSF1. We hypothesised that FN may be a stress-responsive gene and that the differential effects of GA and NOV on FN may be explained by the ability of GA but not NOV to induce an HSF1-mediated stress response. To test this hypothesis, we first demonstrated that our GA treatments were sufficient to induce a stress response in cells (Fig. 4). GA treatments that lead to an increase in FN mRNA and promoter activity also caused an increase in the mRNA levels of known stress-responsive genes *HSP90AA1* (Hsp90alpha), *HSPA1A* (Hsp72), *HSPB1* (Hsp27) relative to the controls *ACTB* (actin) and *PPIA* (peptidylprolyl isomerase A) in both Hs578T and HEK293T cells (Fig. 4A,B). In addition, GA but not NOV caused an accumulation of HSF1 in the nucleus of Hs578T cells (Fig. 4C). These results demonstrate that GA resulted in an activation of the HSF1 mediated stress response in our treated cells, which may explain the increases in FN promoter activity and mRNA upon GA treatment. Consistent with this interpretation, other stress conditions including heat-shock and chemically induced hypoxia (using CoCl_2_) also caused an increase in FN mRNA levels (data not shown).

### FN response to GA relies on HSF1 and Heat Shock Elements (HSEs) in the promoter

**Figure 5:**
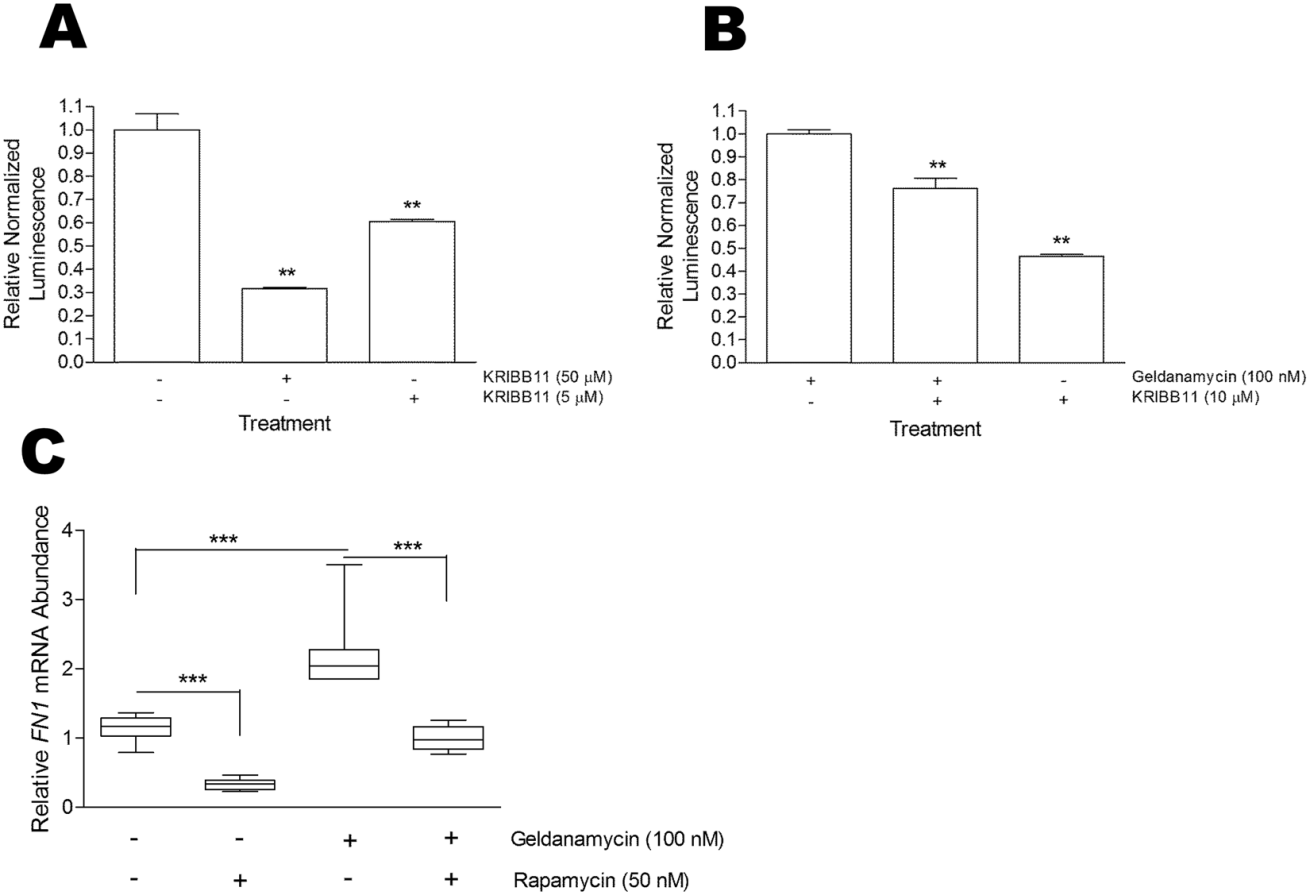
HSF1 inhibition reduces *FN1* expression. HEK293FT cells transfected with the pGL4-pFN1 promoter reporter plasmid and transfection efficiency control pEGFP were treated with or without the HSF1 inhibitor KRIBB11 in the (**A**) absence or (**B**) presence of geldanamycin (GA) for 24 hours. Luminescence values are relative to GFP fluorescence and data shown are representative of 3 biological replicates per treatment with 3 technical replicates per transfection. Statistical analysis was conducted by comparing the treated cells to the DMSO control using one way ANOVA with Bonferroni post-test (**p* < *0.001). (**C**) Hs578T cells were treated for 24 hours with GA (100 nM), Rapamycin (50 nM), both or the DMSO vehicle control. Total RNA was extracted and qPCR was conducted using *PPIA* as a reference gene. Data shown are average mRNA abundance (ΔCt) relative to the DMSO vehicle control from 3 biological experiments conducted in technical triplicates. Statistical analysis was conducted using one way ANOVA with Bonferroni post-test (***p < 0.001).

To determine whether HSF1 was involved in the response to GA, we tested the effect of the HSF1 inhibitor, KRIBB11^39^, on the activity of the FN promoter (Fig. 5). KRIBB11 resulted in a statistically significant reduction in the FN promoter activity when used alone (Fig. 5A) or in combination with GA (Fig. 5B). In addition, we tested the effect of the mTORC inhibitor, rapamycin, on mRNA levels of FN in the presence and absence of GA treatment (Fig. 5C). Rapamycin inhibits HSF1 by blocking phosphorylation of serine 326 which is required for HSF1 transcriptional activity^40^. GA treatment led to an increase in FN mRNA abundance relative to the DMSO control. Rapamycin treatment at sub-toxic levels (data not shown) caused a significant reduction in HSF1 mRNA levels compared to the DMSO control, and significantly inhibited the GA-induced increase in FN mRNA levels. To support these observations, we interrogated published gene expression datasets for changes in FN mRNA levels upon HSF1 knockout or GA treatment. Our analysis demonstrated a reduction in the FN mRNA levels in HSF null MEFs compared to wild type MEFs (Supplementary Figure 1A,B). In addition, GA treatment resulted in a significant increase of FN mRNA levels (similar to increases in mRNA of known stress-responsive genes) in two cell lines upon GA treatment (Supplementary Figure 1C,D). Taken together, these data provided further support for the fact that FN may be a stress responsive gene and that both the basal and stress-inducible expression of FN was regulated at least in part by HSF1.

**Figure 6:**
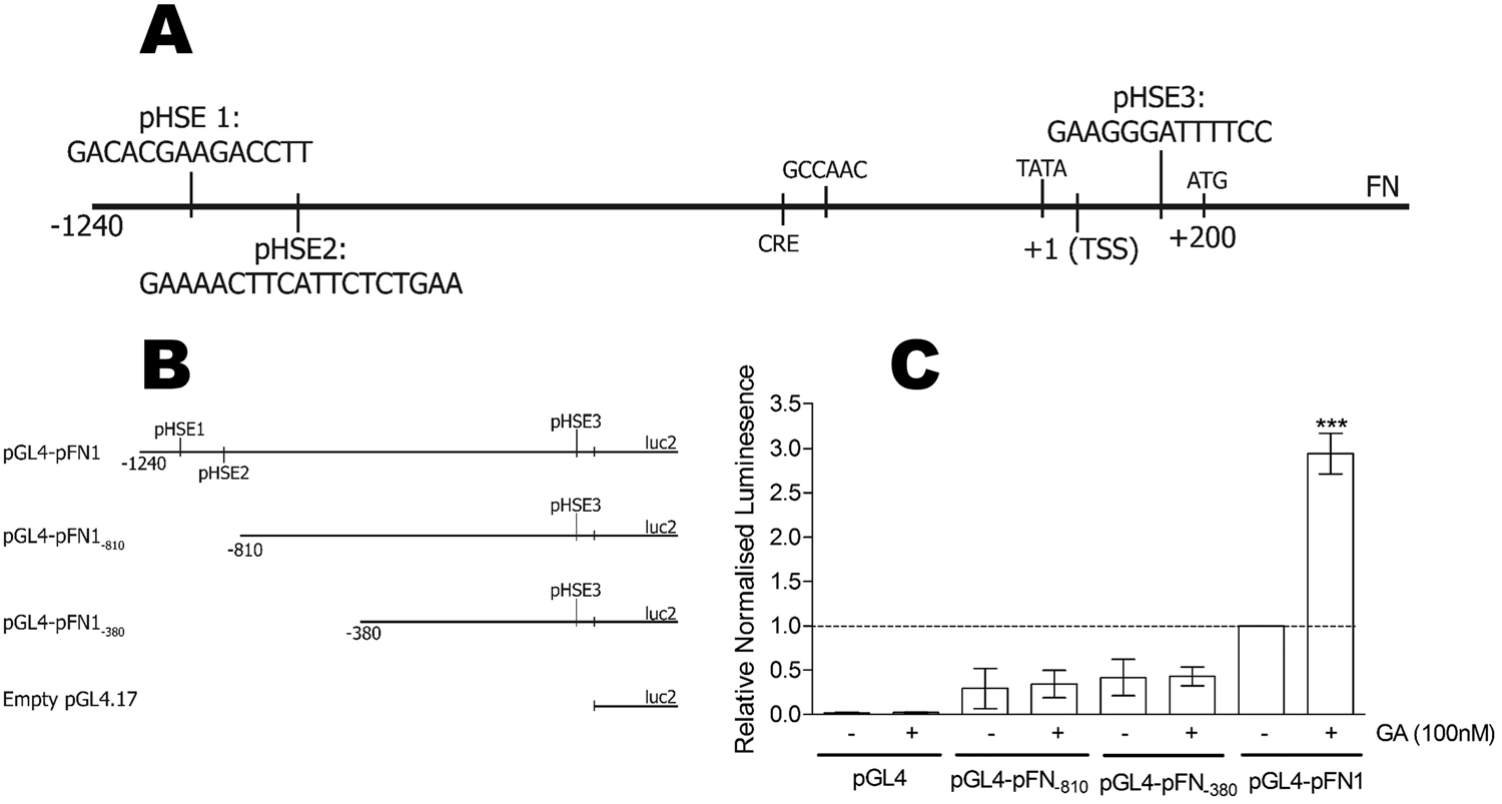
Geldanamycin induced up-regulation of *FN1* is dependent on heat shock elements in the promoter. (**A**) The cloned *FN1* promoter sequence annotated with putative HSEs and key transcription motifs. The CCAAT box (−334 bp) and TATA box (−27 bp) are identified relative to the TSS ( + 1 bp) and start codon ( + 200 bp)^77^. (**B**) Two truncations of the *FN1* promoter were made by PCR amplification and validated by Sanger sequencing. (**C**) HEK293FT cells were transfected with pGL4-pFN1, pGL4-pFN1_−810_, pGL4-pFN1_−380_ or empty pGL4.17 and transfection efficiency control pCAG-HRP-TM and treated with 100 nM GA or DMSO. Relative luciferase values are normalised to the DMSO treated pGL4-pFN1 and are representative of 6 independent transfections per treatment with 3 technical replicates per transfection. Statistical analysis was conducted in comparison to the wild type untreated pGL4-pFN1 treatment using one way ANOVA with Bonferroni post-test (***p* < *0.001).

HSF1 is known to recognise specific sequences, termed heat shock elements (HSE), in the promoters of stress-responsive genes. The genomic sequence encompassing the *FN1* promoter (NCBI: NG 012196.1 from −2000 bp to + 1) was analysed for putative HSEs using transcription factor binding motif algorithms. The TFSearch algoritm^41^ identified three potential HSEs similar to the canonical HSF1 binding motif (nGAAnnTTCnnGAAn)^42^ (Fig. 6A). Two of the putative HSE were upstream of the transcriptional start site (TSS; + 1), namely pHSE1 (−965 bp to −951 bp) and pHSE2 (−806 bp to −787 bp), while the third, pHSE3, was downstream of the TSS ( + 179 bp to + 192 bp) (Fig. 6A).

We generated truncations (pGL4-pFN1_−810_ and pGL4-p FN1_−380_) of the FN promoter to remove the two distal putative HSEs (pHSE1 and pHSE2) to determine whether these sequences were required for the GA-induced increase in *FN1* promoter activity (Fig. 6B). The loss of pHSE1 and pHSE2 resulted in a greater than 50% reduction in the basal activity of the FN promoter (Fig. 6C). In addition, whereas GA induced a strong activation of the full length FN promoter, the loss of pHSE1 and pHSE2 prevented GA-induced promoter activation (Fig. 6C). These data suggested that the DNA region removed by the first truncation (pGL4-p FN1_−810_) contained the motif responsible for the GA-mediated increase in promoter activity. There was also a loss in basal reporter activity in the deletions, indicating that one or more sites involved in the constitutive activity of the *FN1* promoter were also eliminated in the first truncation.

### HSF1 is recruited to the *FN1* promoter upon GA treatment

**Figure 7:**
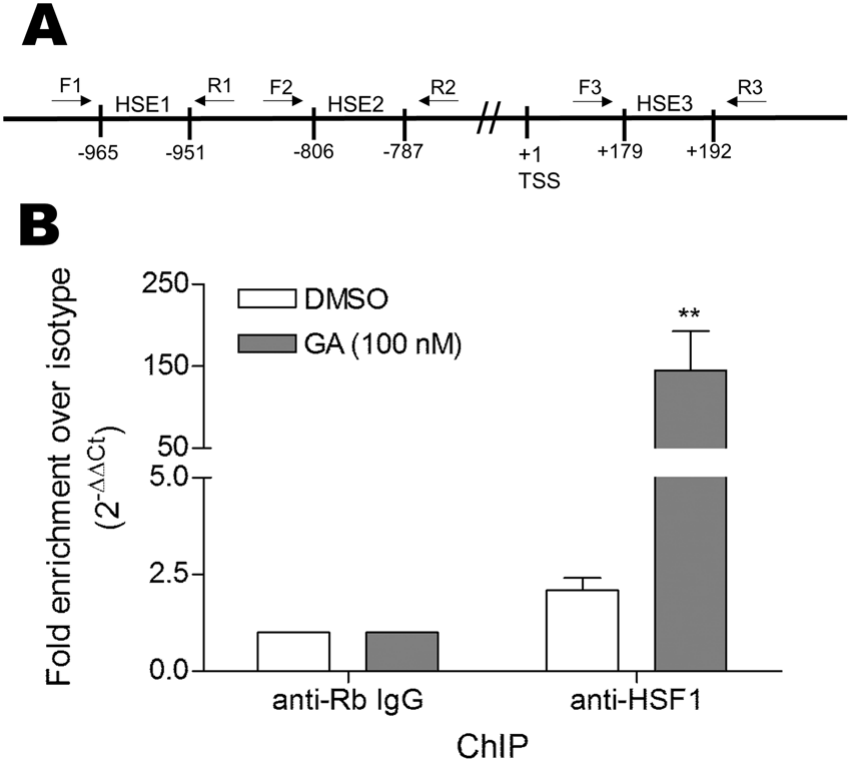
Heat-shock factor 1 occupies a heat shock element on the fibronectin promoter and occupancy increases with geldanamycin treatment. (**A**) Schematic diagram showing the location of putative HSEs in the *FN1* promoter and the location of primer sets used to amplify these regions from ChIP DNA. (**B**) Hs578T cells were treated with either 100 nM GA or DMSO. Chromatin immunoprecipitation with anti-HSF1 antibody or isotype control was conducted using the Imprint UltraChromatin Immunoprecipitation Kit (Sigma-Aldrich) as per the manufacturer’s instructions. The purified DNA was amplified by qPCR using primers F1 and R1 (designed to amplify the region containing HSE1) and the fold enrichment of the putative *FN1* heat-shock element relative to the isotype control and input DNA from the anti-HSF1 immunoprecipitation under the two conditions was determined using the ∆Ct method (2^−ΔΔCt [HSF1/Isotype]^)^43^. Data shown are average fold enrichments from 3 samples, and are representative of 2 independent biological experiments conducted in triplicate. Statistical analysis was conducted using one way ANOVA with Bonferroni post-test (*p* < *0.05) comparing the HSF1 ChIP samples to the relevant isotype ChIP control.

We next tested for direct binding of HSF1 to the putative HSE sequences identified in the *FN1* promoter. Chromatin immunoprecipitation linked to quantitative PCR (ChIP-qPCR) was conducted on both DMSO and GA treated chromatin from Hs578T cells using an antibody against HSF1 or isotype control (Fig. 7). Primer sets were designed to amplify the three promoter regions containing the putative HSEs (Fig. 7A). The primer set targeting HSE1 resulted in an amplicon from both the DMSO and GA treated ChIP reactions, while no amplification was obtained from primer sets 2 or 3 (targeting pHSE2 and pHSE3, respectively). The fold enrichment (2^−ΔΔCt^) of the HSF1 ChIP reaction over the isotype control was calculated from the Ct values obtained using the pHSE1 primer set (Fig. 7B)^43,44^. Binding of HSF1 to the pHSE1 site (−965 bp to −951 bp) was observed for both the DMSO (approximately 2 fold over the isotype control) and GA treated chromatin (approximately 150 fold over isotype control) (Fig. 7B). There was a statistically significant enrichment of HSF1 recruitment to HSE1 in the GA treated cells compared to the DMSO treated control (Fig. 7B). The product obtained from the pHSE1 PCR was confirmed by sequencing to contain the motif matching the predicted pHSE1 sequence (data not shown). These data confirmed that HSF1 bound to the predicted HSE motif between −965 bp to −951 bp on the *FN1* promoter under basal conditions, and that the occupancy of this HSE by HSF1 was increased by GA treatment.

### A subset of other extracellular matrix genes is GA responsive

**Figure 8:**
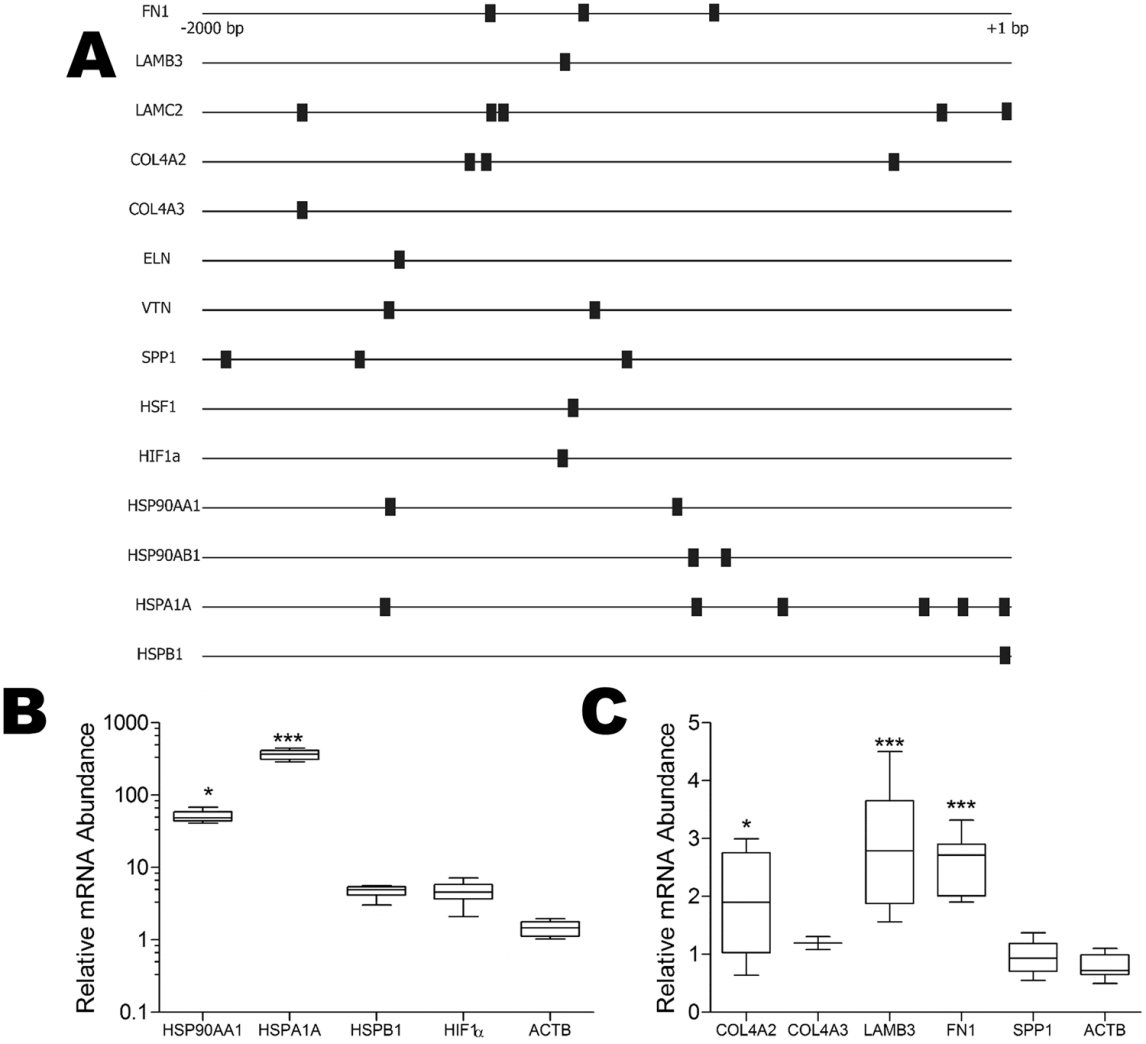
A subset of other extracellular matrix genes are stress responsive. (**A**) The −2000 bp to + 1 bp promoter regions of selected genes were analysed using TFSearch. Fibronectin 1 (*FN1*), laminin β3 (*LAMB3*), laminin γ2 (*LAMC2*), collagen 4 α2(*COL4A2*), collagen 4 α3 (*COL4A3*), elastin (*ELN*), vitronectin (*VTN*), osteopontin (*SPP1*), heat-shock factor 1 (*HSF1*), hypoxia inducible factor α (*HIF1α*), Hsp90α (*HSP90AA1*), Hsp90β (*HSP90AB1*), Hsp72 (*HSPA1A*). The locations of predicted heat-shock elements (HSE) are indicated by black boxes. Hs578T cells were treated with GA 100 nM and the relative mRNA levels from known stress inducible genes (**B**) and a selection of ECM genes (**C**) was assessed by qRT-PCR. Data shown are average mRNA abundance (ΔCt) for genes in the GA treated sample relative to the DMSO treated control (which is 1 in all cases), calculated from 3 independent biological experiments each run in triplicate. Statistical analysis was conducted in comparison to the reference control (ACTB) using one way ANOVA with Bonferroni post-test (*p* < *0.05, ***p* < *0.001).

We next investigated the responsiveness of other ECM proteins to GA treatment. The −2000 bp to + 1 bp promoter regions of known stress inducible genes (*HSP90AA1*, *HSPA1A*, *HSPB1*), stress related transcription factors (*HSF1*, hypoxia inducible factor 1A [*HIF1A*]) and a subset of key extracellular matrix protein genes (laminin beta 3 [*LAMB3*], laminin gamma 2 [*LAMC2*], collagen 4 alpha 2 [*COL4A2*], collagen 4 alpha 3 [*COL4A3*], elastin [*ELN*], vitronectin [*VTN*] and osteopontin [*SPP1*]) were screened for potential HSEs using TFSearch. The locations of predicted HSEs on the gene promoter regions are shown as black boxes (Fig. 8A). At least one putative HSE was predicted in the promoter regions of all the genes analysed (Fig. 8A). GA treatment of Hs578T cells resulted in an increase in mRNA abundance of HSP90AA1 (50-fold), HSPA1A (360-fold), HSPB1 (4.5-fold) and HIF1α (4.5-fold) (Fig. 8B), which is consistent with the stress-inducible nature of these genes. The levels of SPP1 and COL4A3 mRNA did not change after treatment of Hs578T cells with GA, although LAMB3 (3-fold), COL4A2 (2-fold) and FN (2.5-fold) mRNA increased significantly upon GA treatment compared to the DMSO control (Fig. 8C). These data suggest the FN may not be the only ECM gene responsive to GA treatment.

## Discussion

We propose that FN is a stress-responsive gene and its expression is regulated by the major stress-responsive transcription factor, HSF1. GA induced a stress response that increased HSF1 binding to an HSE between positions −965 bp to −951 bp of the *FN1* promoter, leading to upregulation of promoter activity causing an increase in FN1 mRNA, protein and subsequently extracellular matrix. To the best of our knowledge, this is the first report of a link between the stress response, HSF1 and FN expression. We have previously shown that FN is a client protein of Hsp90 and that inhibition using the C-terminal Hsp90 inhibitor novobiocin results in turnover of the FN ECM^29^. We predict that the effects of novobiocin on FN are different to those of GA, because novobiocin does not induce a stress response. GA may also cause some turnover of the FN matrix by inhibiting Hsp90, however this loss of FN is likely to be masked by the induction of FN expression through the activation of HSF1.

A number of human HSF1 target genes have been previously identified using microarray and ChIP-seq analyses. Activation of HSF1 by heat shock results in rapid upregulation and downregulation of thousands of cellular genes, many of them members of the heat shock protein family^45^. In the context of cancer, active HSF1 drives malignancy by regulating transcription of a wide range of genes, many of which are distinct from those induced by heat shock^46^. Analysis of GEO datasets from those studies that included FN, show that FN mRNA levels are reduced in HSF1 null MEFs compared to wild type MEFs^42,47^, and increased in cells treated with GA^48,49^, which together is consistent with our data that FN1 is regulated by HSF1 in response to GA. In addition to FN we showed for the first time that the mRNA levels of two other ECM genes, LAMB3 and COL4A2, increased with GA treatment in the Hs578T cell line. Data from HSF1 null fibroblasts showed a decrease in COL4A2 expression compared to normal fibroblasts (Supplementary Figure 1A). Unfortunately, to the best of our knowledge there are currently no publically available microarray or ChIP-seq studies that have performed a global analysis of the effect of GA on HSF1 transcription.

HSF1-mediated induction of FN, and the potential of parallel regulation for COL4A2 and LAMB3, is of interest because these proteins are major components of the interstitial matrix and the basement membrane^50^, changes in which can lead to disease. In particular, our results have implications for disease in the following contexts: 1) diseases in which HSF1 activation is a hallmark (such as cancer), 2) diseases characterised by increased FN deposition by unknown mechanisms (such as diabetes) and 3), diseases in which GA or other N-terminal inhibitors known to activate HSF1 are to be used therapeutically.

The cancerous environment is often characterised by a number of proteotoxic stresses arising from nutrient and oxygen deprivation, signalling imbalances and mutated oncoproteins^51^ leading to constitutive activation of HSF1. Deregulated ECM dynamics, whether they result in a loss or over deposition of ECM components, are also considered a hallmark of cancer^52^. The deposition of ECM proteins is key to the progression of the metastatic cancers, enabling angiogenesis and promoting cell migration^53^. Some cancer types, such as intestinal and lung carcinoma, exhibited increased FN expression particularly at the invading edge of migrating tumours^54^. In colon cancer, FN levels were positively correlated with TNM stage and increased FN expression was linked with increased malignancy, proliferation and a poorer disease prognosis^55^. High levels of HSF1 activation in tumour cells have been associated with increased cell growth and metastasis^56–58^. It is possible that in cancers with active HSF1, the increased levels of FN have a pro-invasion and survival effect and support cancer progression independent of the surrounding milieu. Interestingly, it has been demonstrated in animal models that treatment with GA analogues caused an increase in the incidence of metastases^59^, which is consistent with the promotion of the adhesion, migration and development of cancer metastases by increased FN levels^60^.

Proteotoxic and oxidative stresses produced by cancers are mirrored in several aspects by nutritional stresses. HSF1 regulation is closely interconnected to nutritional state^61^ and the nutrient sensor mTORC1 has been shown to directly regulate HSF1 activation by phosphorylation^40^. A number of studies have demonstrated that high glucose conditions, such as those observed in diabetes increase the mTORC1 kinase activity and activate HSF1^62,63^. Diabetes is also known to present with pathologies associated with excess ECM protein deposition^63^. These pathologies include retinopathy^64^ and nephropathy^65^ in which thickening of the basement membrane is a hallmark^66^. The mechanism leading to the increased basement membrane is poorly described. Interestingly, the primary components of the basement membrane (laminin, collagen and FN) are the proteins observed in this study to increase with GA treatment. It is tempting to speculate that the HSF1-mediated increase in ECM proteins might be one mechanism to explain the thickening of the basement membrane in high glucose conditions associated with activated mTORC1, HSF1 and diabetes. The use of N-terminal Hsp90 inhibitors in the alleviation of diabetes related symptoms has also been investigated due to the increased levels of insulin sensitivity observed upon HSF1 activation^67^. In light of the findings of our study, it might prove beneficial to investigate any fibrotic effects of treatment regimens using GA analogues in diabetes.

The results of the present study also have implications for the therapeutic use of N-terminal Hsp90 inhibitors, many of which are in clinical trials as anti-cancer agents^68^. We have shown that GA induces FN expression via an HSF1 mediated stress response, and many of the Hsp90 inhibitors currently in clinical trials are thought to activate HSF1^69^. The concentrations used *in vivo* in clinical trials are in the range of 20–100 mg/L/24 h^70–72^. *In vivo* concentrations of the GA analogue 17-AAG are reported to peak at between 1.7–3 µM^73^, 200 fold higher than the 10 nM we have shown to increase the levels of FN ECM *in vitro*. Regardless of the rate at which these compounds are metabolised, large parts of the body may receive low doses of Hsp90 inhibitors. Although these doses will be subtoxic, the data from our study indicate that low GA concentrations are sufficient to increase FN promoter activity and FN protein levels. The result of this might be the development of aberrant ECM dynamics and concomitant pathologies in tissues distant from the tumour site. It would be interesting to observe whether current clinical trials yield results indicating changes to the ECM.

## Methods

### Antibodies

Primary antibodies used were: mouse anti-human fibronectin (Sigma-Aldrich, F0916), rabbit anti-human fibronectin (Sigma-Aldrich, F3648), rabbit anti-human HSF1 (Santa Cruz Biotechnology, H-311), and mouse anti-human GAPDH (Sigma-Aldrich, G8795). Secondary antibodies used were goat anti-mouse IgG conjugated to HRP (Abcam, ab97023), donkey anti-mouse IgG conjugated to DyLight® 488 (Abcam ab96875) and donkey anti-rabbit IgG conjugated to DyLight® 488 (Abcam ab96891).

### Cell culture

The Hs578T breast cancer cell line was purchased from ATCC (HTB-126) and cultured in Dulbecco’s Modified Eagle Medium (DMEM) with 10% (v/v) Foetal Bovine Serum (FBS), 1 mM L-Glutamine, 100 U/mL penicillin, streptomycin, amphotericin (PSA) and 2 mM insulin at 37 °C, with 9% CO_2_. The HEK293FT cell line, which is a highly transfectable clonal isolate derived from human embryonal kidney cells transformed with the SV40 large T antigen (R70007, Thermo Fischer), was a gift from Sharon Prince (University of Cape Town) and was maintained in DMEM with 5% (v/v) FBS, 1 mM L-Glutamine, 0.1 mM non-essential amino acids (NEAA), 1 mM sodium pyruvate and 100 U/mL PSA at 37 °C, with 9% CO_2_.

### Immunofluorescence

A total of 5 × 10^4^ Hs578T cells were seeded on sterile glass coverslips and treated as described in figure legends for 24 h before processing. Cells were washed with sterile phosphate-buffered saline (PBS) (137 mM NaCl, 10 mM Na_2_HPO_4_, 1.76 mM KH_2_PO_4_ 2.7 mM KCl, pH 7.4), fixed in ice-cold methanol for 10 seconds and blocked using 1% (v/v) bovine serum albumin (BSA) in sterile PBS for 45 mins at room temperature. Coverslips were incubated with primary antibodies diluted in 0.1% (v/v) BSA/PBS overnight followed by two washes of 5 mins with 0.1% (v/v) BSA/PBS. Fluorescently conjugated species-specific secondary antibodies in 1% (v/v) BSA/PBS were incubated with coverslips in the dark for 1 hour at room temperature before washing twice for 5 mins with 0.1% (v/v) BSA/PBS. A brief wash with Hoescht-33342 (1 µg/mL) in distilled water was used to stain cell nuclei and coverslips were mounted on to slides using Dako fluorescent mounting medium. Slides were visualised using a Zeiss Axio Vert.A1 fluorescence microscope or Zeiss LSM 780 confocal microscope using ZEN Black software.

### Immunoblotting

Protein samples were prepared from treated cell monolayers or harvested cell pellets using 5 × SDS sample buffer (250 mM Tris-HCl pH 6.8, 10% [w/v] SDS, 30% glycerol [v/v], 5% β-mercaptoethanol [v/v], 0.02% [v/v] bromophenol blue) and separated using gel electrophoresis at 150 V in 1 x SDS-PAGE running buffer (0.25 mM Tris, 192 mM glycine, 1% [w/v] SDS) with a 4% (v/v) stacking gel and an 8% (v/v) resolving gel as per standard protocols^74^. Resolved samples were transferred to nitrocellulose membranes as per standard protocols^75^ in western transfer buffer (13 mM Tris-HCl, 100 mM glycine and 20% [v/v] methanol) at 400 mA for 1 h. Membranes were blocked using 5% [w/v] Blotto solution in 1 x Tris-buffered saline with Tween-20 (TBS-T; 50 mM Tris pH 7.5, 150 mM NaCl, 0.1% [v/v] Tween-20) for 1 hour and incubated with primary antibody in TBS-T at 4 °C overnight. Membranes were washed 3 times for 10 mins each in fresh TBS-T before incubation with HRP conjugated secondary antibodies in TBS-T for one hour at room temperature. Three final 10 min washes in TBS-T were conducted before signal development using chemiluminescent substrate.

### Quantitative real-time PCR

All qPCR primers used were from IDT. A total of 5 × 10^4^ Hs578T cells or 1 × 10^5^ HEK293FT cells per well were treated with compounds or vehicle controls as described in the figure legends. Total RNA was extracted using Direct-zol RNA miniprep kit and equal amounts of total RNA were converted to cDNA using a RevertAid First Strand cDNA Synthesis Kit with Oligo (dT)_18_ primers followed by qRT-PCR with KAPA SYBR FAST qPCR reagents as per the manufacturer’s instructions on a CFX Connect Real-Time PCR Detection System. A number of housekeeping genes were assessed for stability prior to analysis and ACTB and PPIA were selected as controls for all subsequent qPCR assays. The raw qPCR data were analysed to calculate either average normalised fold expression (2^−ΔΔCt^) or mRNA abundance (ΔCt) relative to the control sample (DMSO treated). The ΔCt values were calculated for control and treated conditions according to the equation ΔCt = Ct Target Gene - Ct Reference Gene. The ΔΔCt was calculated according to the equation ΔΔCt = ΔCt Test - ΔCt Control and converted to normalised fold expression using the calculation 2^43^.

### *In silico* promoter analysis

The genomic sequence for the fibronectin gene (*FN1*) (NCBI: NG 012196.1) was retrieved from the ENCODE human genome build 19 (http://genome.ucsc.edu/ENCODE/). The region from 5000 bp upstream (5′−3′) and 1000 bp downstream of the transcription start site (TSS located at 216,211,070 bp of human chromosome 2) was retrieved and TFSearch^41^, used with default settings to identify putative HSEs.

### Luciferase reporter assays

The *FN1* promoter sequence from − 1240 bp to + 100 bp was synthesised by GenScript and cloned into the promoterless pGL4.17 plasmid via the *Nhe*I and *Bgl*II restriction sites to create the pGL4-pFN1 construct. Truncations of the p*FN1* sequence were produced by PCR from pGL4-pFN1 using a common reverse primer (AGATCTCACCAAGTTTGCTTCCC) and distinct forward primers depending on the truncation (for pFN1−810 the forward primer was GCTAGCGAACTCCCGGTACTTAGTAG, while for pFN1−380, the forward primer was GCTAGCGTCTGGATTCTTAACAGCTGC). All plasmids were confirmed by sequencing and endotoxin-free preparations prepared for transfections using the Qiagen Endotoxin free Maxiprep kit according to manufacturer’s instructions. A total of 1× 10^5^ HEK293FT cells were transfected with pGL4-pFN1 (or truncations) and either pCAG-HRP-TM (a gift from Alice Ting; Addgene plasmid # 44441) or pLV-eGFP (a gift from Pantelis Tsoulfas; Addgene plasmid # 36083) as a transfection efficiency control using a 1:1 ratio with X-tremeGENE HP transfection reagent. Cells were treated as described in the figure legends 24 h after transfection, and were incubated in treatment conditions for 24 h before the luciferase and HRP or EGFP activities were assayed, unless otherwise stated. Luciferase activity was measured after incubation with FLAR buffer (40 mM Tricine, 200 µM EDTA, 5.14 mM MgSO_4_, 34 mM DTT, 250 µM ATP and 100 µM D-luciferin)^76^ while conversion of 3,3′,5,5′tetramethylbenzidine (TMB) substrate by HRP was measured by absorbance at 450 nm and EGFP fluorescence was detected by excitation at 485 nm and emission at 525 nm.

### Chromatin immunoprecipitation

Hs578T cells were treated for 24 h with either GA (100 nM) or DMSO, washed once with Hank’s balanced salt solution and lifted using 0.25% (v/v) trypsin-EDTA. Formaldehyde was added to a final concentration of 1% (v/v) and cross-linking was allowed to continue for 10 mins with end-to-end rotation. The reaction was stopped using 0.1 volumes of 1.25 M glycine and the cell suspension collected by centrifugation at 200 RCF, 4 °C for 10 mins. The pellet was washed in cold sterile PBS and lysed in lysis buffer containing 1% (v/v) NP-40, 0.1 M 4-(2-aminoethyl) benzenesulfonyl fluoride (AEBSF) and protease inhibitor cocktail on ice for 20 mins. The lysate was treated with 15 strokes of a 2 mL dounce homogeniser (B pestle) and nuclei pelleted by centrifugation at 2,500 RCF for 5 mins at 4 °C. Nuclei were resuspended in nuclear lysis buffer with 0.1 M AEBSF and protease inhibitor cocktail and incubated on ice for 10 mins. Cross-linked chromatin was sheared using DNAseI at 0.05 U/µg chromatin for 30 mins before the reaction was stopped using 5 mM EDTA. The prepared chromatin was used with the Imprint Ultra Chromatin Immunoprecipitaton Kit as per the manufacturer’s instructions using 2 µg/ml HSF1 antibody or isotype control. The resulting purified DNA was analysed via qPCR using KAPA SYBR FAST qPCR reagents as described previously. The primers used were pHSE1 forward primer F1 (AGTTACACACAAAGCAGAGA) and pHSE1 reverse primer R1 (TTTTCAGGTTGTTCACAGTG). The sequence of the HSF1 ChIP qPCR reaction products was confirmed by Sanger sequencing. The fold enrichment of the HSF1 ChIP over the isotype was calculated from the Ct values obtained. ΔCt was obtained by normalising the Ct values from the HSF1 and isotype ChIP DNA fraction to the input DNA fraction (i.e. ΔCt [normalized ChIP] = (Ct [ChIP] - (Ct [Input] - Log2 (Input Dilution Factor))). The normalized ChIP fraction Ct value was adjusted for the normalised background fraction Ct value (i.e. ΔΔCt [HSF1/Isotype] = ΔCt [normalized HSF1] - ΔCt [normalized isotype]), before calculating the fold enrichment of the HSF1 ChIP over the isotype by linear conversion (i.e. fold enrichment = 2^−ΔΔCt [HSF1/Isotype]^)^43,44^

### Statistical Analysis

All experiments were conducted as a minimum of three independent biological replicates with technical triplicates unless otherwise stated and statistical analyses were conducted using ANOVA with Bonferroni post-test or unpaired two-tailed Students t-tests in GraphPad Prism Version 4 software.

### Data Availability

The datasets generated during and/or analysed during the current study are available from the corresponding author on reasonable request.

## Electronic supplementary material

Supplementary information
