# Supplementary material for: Fibronectin is a stress responsive gene regulated by HSF1 in response to geldanamycin
Source: Sci Rep. 2017 Dec 15;7:17617. doi: 10.1038/s41598-017-18061-y (PMC5732156; doi:10.1038/s41598-017-18061-y)
Supplement: Supplementary file 1 — Supplementary information [file 41598_2017_18061_MOESM1_ESM.pdf]

Fibronectin is a stress responsive gene regulated by HSF1 in response to geldanamycin

Karim Colin Hassan Dhanani, William John Samson, Adrienne Lesley Edkins*

Biomedical Biotechnology Research Unit (BioBRU), Department of Biochemistry and Microbiology, Rhodes University, Grahamstown, 6140.
*Corresponding author a.edkins@ru.ac.za

Supplementary data file

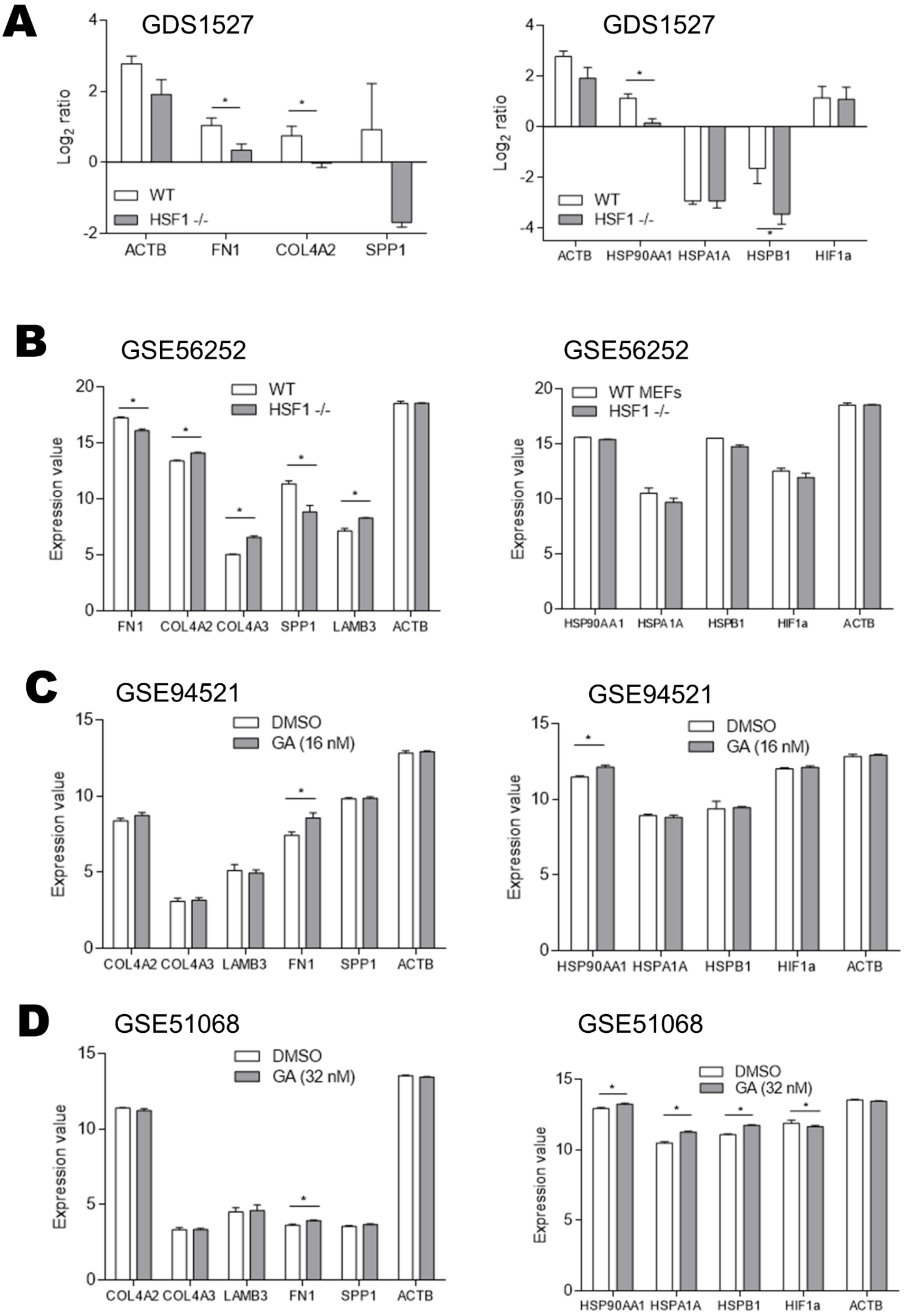
A
GDS1527
Log2 ratio
WT
HSF1 -/-
ACTB
FN1
COL4A2
SPP1
GDS1527
Log2 ratio
WT
HSF1 -/-
ACTB
HSP90AA1
HSPA1A
HSPB1
HIF1a
B
GSE56252
Expression value
WT
HSF1 -/-
FN1
COL4A2
COL4A3
SPP1
LAMB3
ACTB
GSE56252
Expression value
WT MEFs
HSF1 -/-
HSP90AA1
HSPA1A
HSPB1
HIF1a
ACTB
C
GSE94521
Expression value
DMSO
GA (16 nM)
COL4A2
COL4A3
LAMB3
FN1
SPP1
ACTB
GSE94521
Expression value
DMSO
GA (16 nM)
HSP90AA1
HSPA1A
HSPB1
HIF1a
ACTB
D
GSE51068
Expression value
DMSO
GA (32 nM)
COL4A2
COL4A3
LAMB3
FN1
SPP1
ACTB
GSE51068
Expression value
DMSO
GA (32 nM)
HSP90AA1
HSPA1A
HSPB1
HIF1a
ACTB

Supplementary Figure 1: Expression levels of ECM and known stress-inducible genes from published gene expression datasets.

Data shown are average values for multiple probes against the relevant gene. GEO datasets were accessed via the NCBI database using the following accession codes: A) GDS1527 HSF+/+ MEF compared to HSF-/- MEF[42], B) GSE56252, HSF+/+ MEF compared to HSF-/- MEF[47], C) GSE94521, H9-Gll1hESC treated with DMSO or 16 nM GA[49], D) GSE51068, OCI-LY3 B-cell lymphoma treated with DMSO or 32 nM GA[48]. Statistical significance was determined by unpaired two-tailed Student t-tests comparing the control to the test sample for each gene (*p<0.05).
